# A Comparative Study of the Bioavailability of Fe, Cu and Zn from Gluten-Free Breads Enriched with Natural and Synthetic Additives

**DOI:** 10.3390/foods9121853

**Published:** 2020-12-12

**Authors:** Anna Rogaska, Julita Reguła, Joanna Suliburska, Zbigniew Krejpcio

**Affiliations:** Department of Human Nutrition and Dietetics, Poznan University of Life Sciences, Wojska Polskiego St. 31, 60-624 Poznan, Poland; anna.rogaska89@gmail.com (A.R.); joanna.suliburska@up.poznan.pl (J.S.); zbigniew.krejpcio@up.poznan.pl (Z.K.)

**Keywords:** bioavailability, minerals, iron, copper, zinc, rats, gluten-free breads, enrichment

## Abstract

The aim of this study was to compare the bioavailability of iron, copper and zinc from newly designed gluten-free breads enriched with natural and synthetic additives. The study was conducted on rats with induced Fe, Cu and Zn deficiency. The nutritional intervention with diets supplemented with a 70% addition of gluten-free breads enriched with natural additives and organic compounds to the control diet AIN-93M lasted 40 days. After the intervention, the rats were euthanized, the organs were collected and their mineral content was measured. Chemical analysis of diets with the addition of fortified gluten-free breads showed significantly higher amounts of iron, zinc and copper in diets with the addition of fortified breads compared to diets with the addition of unenriched breads. The type of additives did not influence the amount of minerals in diets. It is necessary to conduct further research to explain the interactions of ingredients and the factors affecting the bioavailability of Fe, Cu and Zn from gluten-free breads in order to obtain a product with a high bioavailability of these ingredients.

## 1. Introduction

The constantly deteriorating quality of food products and the increasing occurrence of food intolerances in society poses challenges to dieticians, food technologists and producers. Consumers have ever higher demands regarding food products. In addition to palatability and general desirability, the composition of the product and the functional additives play an increasingly important role.

Gluten intolerance and celiac disease are increasingly common diseases that affect both children and adults [1,2] It is estimated that about 1–2% of the European population has a problem with gluten intolerance [3]. In these people, after consuming gluten, and more specifically its gliadin fraction, the intestinal villi are damaged and atrophied, which, apart from digestive system ailments, leads to impaired absorption of nutrients. As a result, patients struggle with frequent deficiencies of vitamins and minerals such as vitamin B12, iron, zinc and copper. The only effective method of fighting disease is to follow a gluten-free diet for life [4,5]. In order to meet their expectations and needs, the baking industry makes every effort to produce gluten-free bread with the best composition, and structural and organoleptic properties [2]. Producers of food for special nutritional uses, which include gluten-free bread, are trying to produce a product similar to the traditional one, both in terms of its composition and nutritional value [2]. It should be noted that gluten-free products always contain more carbohydrates, fat and saturated fatty acids than their gluten counterparts [5]. Gluten-free bread is mainly produced from rice and buckwheat flour, as well as corn and sorghum [2,6]. Rice flour, which has hypoallergenic properties, is the most delicate and is easy to digest. Its disadvantage, however, are evident when it is used when baking yeast products, as the lack of gluten results in less yeast growth [7]. Such flours do not provide a good structure for the bread, which becomes very brittle, and does not last as long [2]. Gluten-free bread is also characterized by a lower content of minerals than traditional bread, which in many countries, e.g., in Great Britain, is additionally fortified with minerals commonly found in deficiency. These ingredients include calcium, magnesium and iron [3,6]. However, the enrichment of bread must be carried out carefully, as research indicates the accumulation of metals in the enterocytes of people suffering from celiac disease [8,9]. An important element is also the bioavailability of minerals, which depends on many factors such as food composition or individual characteristics, e.g., age, sex and physiological condition. The source of the mineral component and the quantitative ratio of the individual elements to each other are equally important [10]. Iron in heme form is absorbed to about 20–30%, while in non-heme form it depends strongly on other food components. Bioavailability is inversely related to the systemic pool. Vitamin C, hydrochloric acid, sorbitol, ethanol, lactic acid and tartaric acid affect the increase in absorption. Vitamin E, phytates, polyphenols, fiber, egg albumin, casein, legume proteins, copper, calcium, magnesium carbonate, tea, coffee and cigarettes contribute to the decrease in absorption [11]. Copper absorption is on average 35–40% and increases with lower consumption of this ingredient. Increased absorption is observed in the presence of fructose and animal protein. The absorption deteriorates in the presence of zinc, iron, cadmium and molybdenum, high doses of vitamin C, vegetable protein, phytates, sulfur compounds, ascorbic acid and antacids [12]. Zinc is absorbed on average by 20–40%, preferably from animal products. Vitamin B6, D and hydrochloric acid have an influence on the increase in absorption. The absorption is reduced by phytates, fiber, alcohol, oxalates, copper, iron, calcium and phosphates [13,14].

It is increasingly common to enrich gluten-free products with seeds, milk, fruit or other plant additives in order to increase the mineral and vitamin content [6,12,15]. Bread made from quinoa and linseed is characterized by a very good ratio of polyunsaturated to saturated fatty acids and a low content of trans acids. These products are also recommended due to the content of *n*-3 acids. The addition of amaranth, quinoa and buckwheat provides a higher level of protein, fat and minerals compared to bread baked with rice or corn flour [16]. Enriching gluten-free bread with teff increases the carbohydrate, dietary fiber, essential amino acid, iron, calcium, copper and zinc content. However, due to the unacceptable taste properties (an unwanted bitter aftertaste), it must not be added in an amount greater than 10% of the bread weight [17]. The above research confirms the need for further work on additives for gluten-free bread. It is also important to analyze whether the use of natural additives increases the bioavailability of minerals from products in relation to bread enriched with synthetic ingredients. Therefore, the aim of the study was to assess the bioavailability of Fe, Cu and Zn from breads enriched with natural and synthetic additives.

## 2. Materials and Methods

### 2.1. Bread Recipe

The research material consisted of gluten-free breads designed to obtain the highest possible content of deficient minerals while maintaining a good structure and good taste of the bread. The breads were produced in two variants: light—rice (R) and dark—buckwheat (B), with the same amounts of additives (33% rice or buckwheat flour, 17% corn starch, 33% potato flour, 4% pectin each and yeast, 5% sugar, 1% salt and 3% rapeseed oil). Additional poppy seeds and milk were added to the breads in the amounts of 7% and 4%, respectively, reducing the amount of all rice/buckwheat flour to 30%, corn starch to 14% and potato flour to 29% (RM—rice bread with milk and poppy seeds, BM—buckwheat bread with milk and poppy seeds). In order to enrich the breads with synthetic additives (ROm and BOm breads), the addition of organic compounds was used in the following amounts: zinc gluconate 455.67 g/mol, iron (II) gluconate 446.14 g/mol, copper (II) gluconate 453.84 g/mol.

**Figure 1:**
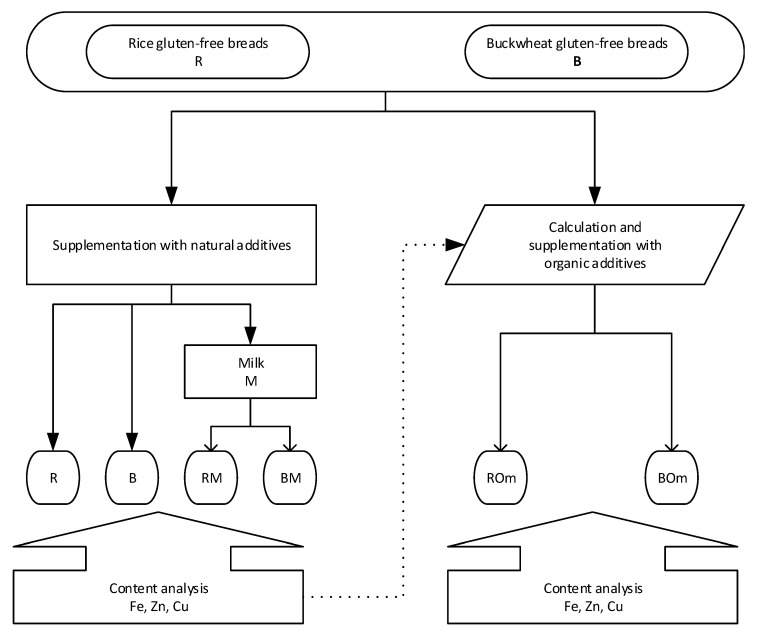
The stages of bread design. Breads: R—light, rice, B—dark, buckwheat, RM and BM—breads with milk and poppy seeds, ROm and BOm—breads with synthetic additives.

The breads with the addition of organic compounds were designed so that the content of minerals was similar to the corresponding breads with natural additives. The stages of bread design are shown in Figure 1.

The breads were baked using the two-phase sourdough fermentation method. The dough was allowed to rise in an oven for 40 min at 35 °C, then it was baked for 23 min at 195 °C. After baking, the breads was dried and ground.

### 2.2. Experimental Design

**Figure 2:**
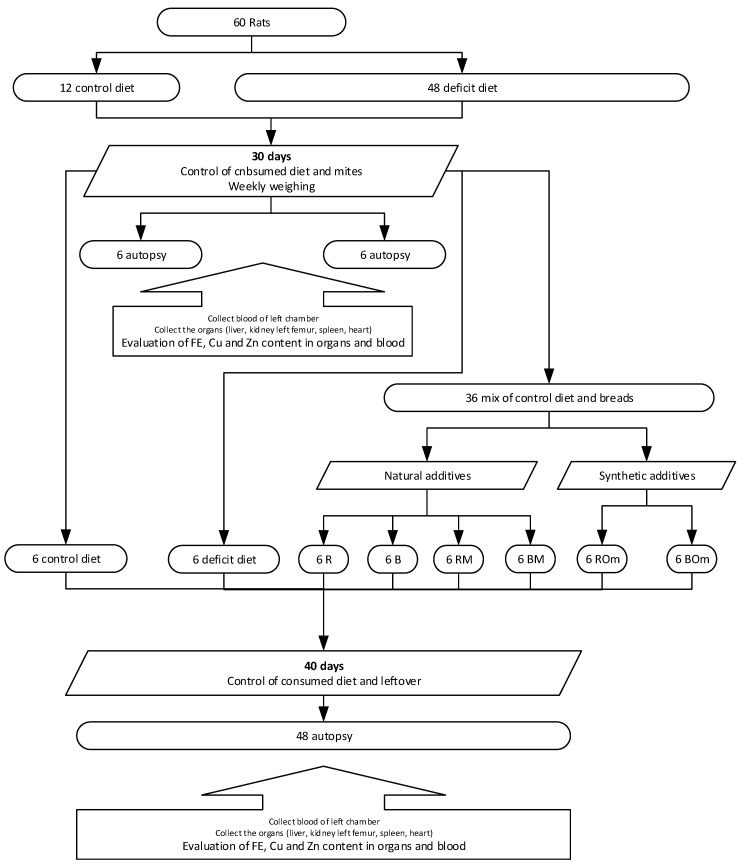
The in vivo experimental model.

The study was performed on 60 male Wistar rats with an average body weight of 265.8 g ± 36.8 g. The study received a positive opinion from the local ethics committee No. 888/11. After initial adaptation, a 30-day period of iron, zinc and copper deficiency was applied using a modified AIN-93M diet composed of: 200 g kg^−1^ Caseine (a manufacturer of dairy products in Murowana Goslina), 70 g kg^−1^ rapeseed oil, 100 g kg^−1^ saccharose, 530 g kg^−1^ wheat starch (Celiko Poznan), 50 g kg^−1^ potato starch (a manufacturer of potatoes in Iława), 3 g kg^−1^ l-cystine (Sigma-Aldrich, Darmstadt, Germany), 2 g kg^−1^ choline (Sigma-Aldrich Germany), 35 g kg^−1^ mineral mix AIN-93-MX [18] (without Fe, Zn and Cu in the mineral mix), 10 g kg^−1^ vitamin mix AIN-93-VX [18]. After this period, the levels of Fe, Cu and Zn in the peripheral blood and selected organs (liver, spleen, heart, kidney and left femur) were determined in 12 randomly selected rats. Then the remaining rats were divided into 8 equal groups, one of which remained on a deficit diet, and one on the control AIN-93M diet, while the remaining rats were supplemented with a 70% addition of gluten-free breads enriched with natural additives and organic compounds to the control diet AIN-93M in 6 different variants (Figure 2). The nutritional intervention lasted 40 days, after which the rats were anaesthetized with a mixture of carbon dioxide and air, peripheral blood was collected from the left ventricle of the heart, and organs (left femur, kidneys, heart, liver and spleen) were collected for iron, zinc and copper content. Throughout the period of the intervention, the amount of food consumed and left behind was monitored, and the body weight of the animals was checked every week. The food efficiency ratio (FER) was calculated as the increase in weight after consuming 100 g of the diet (weight gain [g]/food intake [g] × 100). The animals had constant access to water and food ad libitum. The room in which the animals were kept was air-conditioned, with a constant temperature of 21 ± 1 °C, without access to daylight, with an automatically changing day/night cycle every 12 h, and a relative humidity of 55–65%.

### 2.3. Analysis of Fe, Cu and Zn in Biological Material and Diets

The iron, copper and zinc content of the tissues was determined following digestion of around 0.5 g of each tissue in 65% (*w*/*w*) spectra pure nitric acid (Merck, Kenilworth, NJ, USA) in a microwave digestion system (MARS 5, CEM Corp., Stallings, NC, USA). After digestion all samples were diluted in deionized water. Thereafter, the concentrations of minerals in the mineral solutions were measured using the flame atomic absorption spectrometry method (AAS-3, Zeiss spectrometer). The accuracy of the method was verified with certified reference material (bovine liver-trace elements, NIST-1577C, CERT) with the following results for Fe, Cu and Zn: 94%, 102% and 95%, respectively [19].

Iron, copper and zinc concentrations in serum were established by a commercial laboratory with the use of the ROCHE Module COBAS 6000 analyzer.

The 2 g samples of diets were ashed in a muffle furnace at 450 °C until complete mineralization and then dissolved in 1 moL/L nitric acid (Merck). The mineral content of the samples was determined by flame atomic absorption spectrometry (AAS-3, Zeiss spectrometer), after appropriate dilution with deionized water. The methods were validated by a simultaneous analysis of the reference material (Brown Bread BCR191, Sigma-Aldrich), with the accuracy for Fe, Zn and Cu of 94%, 95% and 104%, respectively [20].

The mineral contents in the tissues and in the diets were measured at wavelengths of 248.3 nm for Fe, 213.9 nm for Zn, and 324.8 nm for Cu. Deionized water and acid-washed glassware were used in this study.

Analysis of variance was used for the statistical analysis of the results, and intergroup differences were assessed with Tukey’s test. A difference of variables between groups of *p* < 0.05, was considered as significant. Calculations were performed in Statistica 13.1.

## 3. Results

Table 1 shows the general nutritional parameters of rats after the end of the experiment. There were no significant differences in the amount of the diet consumed, the starting or final weight in any group. On the other hand, a significantly higher difference in body weight was observed in the group of rats fed the diet with the addition of breads with milk and poppy seeds compared to the diets with breads without this addition. Consequently, the FER index was also significantly higher in these groups. There were no significant differences in organ weights for either group. The type of breads did not affect any of the parameters analyzed.

Table 2 presents the content of the minerals tested in the diets. Both rice and buckwheat breads showed a significantly higher content of all the minerals examined in breads with additives compared to breads without additives.

Table 3 shows the amount of iron consumed by animals and its content in selected organs. Significantly higher consumption of this mineral was found in the M and Om diets compared to diets with no additives. There was no significant difference in iron intake in rats fed the M and Om diets. A significantly higher iron consumption was also observed in rats fed diets with buckwheat breads in all its variants compared to diets containing rice breads. There were no significant differences in the amount of femur iron for either group. In the kidneys, on the other hand, a significantly lower iron content was observed in the groups of animals fed diets with rice breads with natural additives, and significantly, it was lowest in the groups fed diets with rice breads with a synthetic addition compared to the group fed diets with breads without any addition. In the case of iron content in the spleen, its amount was highest in the group fed with rice breads without additives and lowest in the group fed a diet with natural additives. In the group of animals fed diets with buckwheat breads, the highest iron content was found in the spleens of rats fed diets with Om breads, and the lowest in diets with breads without additives. There were no significant differences in the iron content in the rat hearts for any of the groups. Analyzing the iron content in the liver, it can be noticed that both for the group of animals fed diets with rice and buckwheat breads, the highest content of this nutrient was recorded in diets with no additives, while the lowest was in diets with breads with natural additives. The highest significant iron content in the blood was observed in the diets with breads with natural additives, while the lowest was in diets with breads with synthetic additions.

Table 4 shows the results concerning the zinc content in the organs of the animals tested. Significantly higher consumption of this mineral was observed in rats fed diets with the addition of M and Om breads. Significantly higher zinc consumption was also demonstrated for the groups fed diets with buckwheat breads compared to rice breads. A significantly higher zinc content was observed in the femur of rats fed diets with rice breads enriched with natural and synthetic additives compared to breads without additives. A similar relationship was not demonstrated for the groups fed with buckwheat breads, although an effect of the type of flour (rice or buckwheat) on the amount of Zn in the femur was noticed in the case of the groups fed with M and Om breads. There were no significant differences in the zinc content in kidneys and spleens for any of the groups. Significantly higher Zn content was demonstrated in the hearts of rats fed diets with M buckwheat breads. In this case, the difference was also between rice and buckwheat breads. Significantly lower zinc content was found in the livers of rats on a diet with rice breads with a natural additives. Significant differences were also noted between rice and buckwheat breads without additives and with natural additives. In the blood of the animals tested, a significantly higher level of zinc was shown for those fed with a diet with the addition of breads enriched with natural ingredients, both for rice and buckwheat breads. However, no differences were found between rice and buckwheat breads.

Table 5 presents the results concerning the content of copper in the organs of the animals tested. Significantly higher consumption of this ingredient was shown for diets with both natural and synthetic additives. In addition, a higher copper intake was shown for diets with no additive buckwheat breads compared to rice breads. A significantly higher level of copper was shown in the femur of rats fed the diet with breads without additives, as well as in rice breads compared to buckwheat without additives. A significantly higher content of the investigated mineral component in the kidneys of rats was shown when fed diets with breads enriched with natural additives, both for rice and buckwheat breads. In spleens, the highest copper content was recorded for diets with rice breads enriched with synthetic additives, while in buckwheat breads, the lowest value was found for diets with breads with natural additives. In hearts, the highest copper content was found for rice breads with synthetic additives and buckwheat breads with natural additives. In the rats’ livers, the highest copper content was obtained in rats fed diets with rice breads without additives. There was also a difference between rice and buckwheat breads. There were no significant differences in the level of copper in the blood of the animals tested depending on the additives. On the other hand, in buckwheat breads without additives, this level was higher than in rice breads.

In Table 6, the content of iron, zinc and copper in the organs of animals was converted into the consumption of these components in the diet. The iron content in the femur of rats on a diet with rice breads with synthetic additives was the lowest. However, in buckwheat breads it was the highest in diets with breads without additives. A difference was also shown between buckwheat and rice breads for breads without additives. In the kidneys, a significant difference was found only for rice breads with synthetic additives. In rats’ spleens, the highest copper content was found for rice breads without additives and buckwheat breads with the addition of breads with synthetic additives. In hearts, however, the highest iron content was found for diets with buckwheat and rice breads without additives. There were also differences between the types of bread for each additive. Similarly, the highest content of this nutrient was found in the livers of rats on a diet with no additives. Significantly, the lowest blood iron level in terms of consumption was demonstrated for rats fed a diet with breads with synthetic additives, both for rice and buckwheat breads. In this case, differences between the types of bread were also shown. Indeed, the highest zinc content in the femur, kidneys, spleen, heart and liver was shown for rats fed a diet with no additions for both rice and buckwheat breads. There were also differences between the types of bread for each of the variants used for the femur and spleen. For the kidneys and the heart, the difference between the types of bread was demonstrated for breads with additives, while for the liver, it was for breads without additives. There was no significant difference in the level of zinc in the blood compared to the additives used, but higher levels of zinc in rice breads than in buckwheat were found for each of the variants. When calculating the content of copper in organs, a significantly higher level of this component was noticed in the femur, heart, liver and blood of rats fed a diet with breads without additives, both for rice and buckwheat breads. Moreover, for rice breads, these levels were significantly higher compared to buckwheat breads. For the kidneys, significantly lower copper levels were found for diets with the addition of breads with synthetic additives, both for rice and buckwheat breads. In the spleen, the highest levels of copper were recorded for rice breads with synthetic additives, and the lowest for buckwheat breads with natural additives.

## 4. Discussion

The bioavailability of minerals is one of the most important features of gluten-free products. In addition to organoleptic features, it is the bioavailability of deficient ingredients that draws particular attention to consumers struggling with the problem of gluten intolerance [21]. However, it is extremely difficult to obtain gluten-free bread with high bioavailability of minerals while maintaining the appropriate structural and taste characteristics. Research shows that this is a topic that is more and more often taken up by scientists dealing with functional and dietary food, although the overwhelming majority of literature reports still focus on improving the structural and rheological characteristics of gluten-free bread [6,15].

The first attempts to enrich bread with minerals were reported in 2003 by a British–Dutch company [16]. Since then, the ingredients with which the bread is enriched and the methods used have been constantly improved [12]. Rice, buckwheat and corn flour are most often used for baking gluten-free bread [15,16]. On the other hand, amaranth and quinoa, which have the highest content of minerals among gluten-free cereals, are commonly used to enrich bread [22,23]. Research proves that gluten-free products are characterized by low bioavailability of minerals [10]. Despite the addition of products with a high content of minerals to bread, it is difficult to obtain their high bioavailability [12,15]. Many studies show a number of gluten-free products enriched with ingredients with a high content of iron, copper or zinc, but they do not verify their actual availability for tissues and organs [23,24]. As shown by the present studies, the addition of new products to bread does not guarantee a higher content of iron, copper and zinc in the rat organs, taking into account the amount of these minerals consumed. In Great Britain, bread producers are obliged to enrich it with minerals commonly found in deficiency. However, this does not apply to gluten-free bread. Research shows that less than 30% of gluten-free bread produced in the UK is fortified with calcium and iron. Hence, the Reference Nutrient Intake coverage for iron in people consuming gluten-free bread is only 11–19% per serving [3].

The most important problem with adding minerals to food is their interaction. Studies show that iron, copper and zinc are antagonistic. The addition of zinc inhibits the absorption of copper, while the addition of iron inhibits the absorption of zinc. Some authors argue that this only happens when iron is dissolved in water [25]. Studies show worse zinc absorption when the iron-to-zinc ratio in the product exceeds the 25: 1 ratio, which also limits the addition of individual ingredients to the products [14].

The addition of copper to food is usually done with copper gluconate and copper sulfate. Zinc gluconate is more stable, while copper sulfate is cheaper and more hygroscopic. However, there are no studies confirming which of them is characterized by the better bioavailability of copper [25].

Research carried out on the American population shows that zinc in healthy people is consumed at the level of about 60–80% of the daily requirement, which, with low bioavailability, gives about 30% of the amount necessary for use by tissues [14]. Bones are a good marker of zinc bioavailability. In the blood, 10–20% of zinc is concentrated in the plasma, the rest in red blood cells. Brazilian Ultra Rice research showed high bioavailability of zinc from zinc oxide-fortified rice compared to ferric pyrophosphate and thiamine mononitrate in 40 Wistar rats. However, the authors talk about the low solubility of zinc oxide, which may hinder the bioavailability of other minerals [14]. Zinc sulfate is characterized by better solubility and comparatively good bioavailability. Both compounds, and also zinc gluconate, are among the 5 approved for food fortification by GRAS (Generally recognized as safe by United States Food and Drug Administration). Studies conducted in Mexico recommend the addition of zinc at the level of 20–50 mg/kg of flour and copper at the level of 1.2–3.0 mg/kg of flour in order to ensure their proper amount in the body [25].

In the authors’ own research, it was possible to obtain statistically higher levels of all the minerals analyzed, both in breads enriched with natural and with synthetic ingredients. Importantly, there were no significant differences between the fortified breads. This bread enrichment translated into higher levels of minerals in some organs (iron in the spleen and blood, zinc in the heart and blood, copper in the kidneys, spleen and heart).

## 5. Conclusions

The content of iron, zinc and copper in the gluten-free breads designed in this study significantly influenced their content in selected organs and blood of rats. When analyzing fortified bread, it can be noted that generally higher bioavailability of iron and copper was obtained from bread with synthetic additives, and zinc from bread with natural additives.

Additional research on organic and inorganic products and compounds is needed, as well as on their interactions, which may have a better impact on the bioavailability of minerals found in deficiency in people struggling with food intolerances, including gluten intolerance.

## Figures and Tables

**Table 1:** General nutritional parameters of rats.

Ingredients	Type of Bread	Without		M		Om	
**Diet consumed [g]**	R	849.4 ± 86.4	a	890.6 ± 58.3	a	920.3 ± 61.2	a
	B	862.5 ± 99.4	a	883.5 ± 80.3	a	880.9 ± 81.4	a
**Initial weight [g]**	R	243.8 ± 32.8	a	269.2 ± 43.1	a	268.7 ± 37.7	a
	B	268.7 ± 43.6	a	269 ± 41	a	268.5 ± 36	a
**Final weight [g]**	R	307 ± 42.3	a	359.5 ± 37.5	a	336.7 ± 37.8	a
	B	329.5 ± 49	a	353.8 ± 42.9	a	329 ± 36	a
**Weight gain [g]**	R	63.25 ± 15	a	90.33 ± 13.89	b	68 ± 21.67	a
	B	60.83 ± 10.38	a	84.83 ± 13.26	b	60.5 ± 15.2	a
**FER = weight gain [g]/food intake [g] × 100**	R	6.33 ± 1.42	a	8.58 ± 1.67	b	6.41 ± 2.14	a
	B	5.93 ± 1.05	a	7.94 ± 1.08	b	5.71 ± 1.49	a
**Femur mass [g]**	R	0.743 ± 0.072	a	0.784 ± 0.084	a	0.78 ± 0.071	a
	B	0.762 ± 0.072	a	0.776 ± 0.066	a	0.774 ± 0.073	a
**Kidney mass [g]**	R	1.98 ± 0.4	a	2.3 ± 0.23	a	2.23 ± 0.28	a
	B	2.14 ± 0.3	a	2.22 ± 0.13	a	2.25 ± 0.29	a
**Spleen mass [g]**	R	0.505 ± 0.07	a	0.503 ± 0.019	a	0.507 ± 0.043	a
	B	0.498 ± 0.042	a	0.527 ± 0.039	a	0.513 ± 0.055	a
**Heart mass [g]**	R	0.966 ± 0.137	a	1.071 ± 0.103	a	1.007 ± 0.067	a
	B	1.021 ± 0.12	a	1.04 ± 0.082	a	0.991 ± 0.1	a
**Liver mass [g]**	R	9.5 ± 1.31	a	10.85 ± 1.23	a	9.95 ± 1.09	a
	B	9.94 ± 1.16	a	10.23 ± 0.94	a	9.64 ± 0.75	a

a, b indicates statistical differences between additives (Without, M—milk and poppy seeds, Om—synthetic additives) *p* < 0.05. FER—food efficiency ratio.

**Table 2:** Content of minerals in diets with gluten free breads [mg/100 g].

Minerals	Type of Bread	Without		M		Om	
**Fe**	R	2.27 ± 0.03	a *	2.79 ± 0.04	b *	2.88 ± 0.03	b *
	B	2.88 ± 0.09	a *	3.43 ± 0.08	b *	3.51 ± 0.02	b *
**Zn**	R	2.07 ± 0	a *	2.42 ± 0.13	b *	2.27 ± 0.15	ab *
	B	2.49 ± 0.04	a *	3.45 ± 0.24	b *	3.28 ± 0.01	b *
**Cu**	R	0.32 ± 0.01	a *	0.45 ± 0.04	b	0.42 ± 0.03	b
	B	0.38 ± 0.01	a *	0.51 ± 0.4	b	0.47 ± 0.02	b

a, b indicate statistical differences between additives (Without, M, Om) *p* < 0.05. * indicates statistical differences between the types of bread (RM—rice bread with milk and poppy seeds, BM—buckwheat bread with milk and poppy seeds) *p* < 0.05.

**Table 3:** Iron content in organs.

Ingredients	Type of Bread	Without		M		Om	
Fe consumed with diet [g]	R	19.28 ± 1.96	a *	24.85 ± 1.63	b *	26.5 ± 1.76	b *
	B	24.84 ± 2.86	a *	30.3 ± 2.75	b *	30.92 ± 2.86	b *
Fe in femur [µg/g d.m.]	R	111.5 ± 26.5	a	123.6 ± 17.7	a	100.9 ± 15.7	a
	B	125.1 ± 11.9	a	104.3 ± 13.7	a	122.8 ± 8.7	a
Fe in femur [µg]	R	53.42 ± 13.68	a	60.97 ± 8.62	a	49.51 ± 7.95	a
	B	59.09 ± 10.78	a	51.16 ± 7.16	a	59.99 ± 9.67	a
Fe in kidney [µg/g d.m.]	R	285 ± 59.3	a	233.5 ± 22.6	ab	207.6 ± 28.2	b
	B	234.7 ± 39.8	a	247.3 ± 37.2	a	210.5 ± 32.5	a
Fe in kidney [µg]	R	130.3 ± 32.5	a	132.3 ± 7.6	a	105.6 ± 17.1	b
	B	119.7 ± 37.7	a	128.7 ± 24.5	a	116.1 ± 17.2	a
Fe in spleen [µg/g d.m.]	R	2014 ± 660	a	1336 ± 329	b	1766 ± 248	a
	B	1760 ± 464	a	1851 ± 329	a	2352 ± 255	b
Fe in spleen [µg]	R	236.7 ± 67.1	a	156.2 ± 34.2	b	179.8 ± 25	ab
	B	175.8 ± 51.2	a	220.7 ± 65	ab	279.1 ± 49.3	b
Fe in heart [µg/g d.m.]	R	316.4 ± 15.7	a	329.1 ± 145.6	a	397.9 ± 136.8	a
	B	294.7 ± 52.5	a	353.3 ± 91	a	320.3 ± 30.3	a
Fe in heart [µg]	R	64.09 ± 11.39	a	69.29 ± 8.25	a	70.87 ± 6.55	a
	B	68.6 ± 9.67	a	66.81 ± 6.67	a	71.03 ± 10.74	a
Fe in liver [µg/g d.m.]	R	958.9 ± 91.3	a	491.1 ± 122.6	b	724.1 ± 133.3	c
	B	802.2 ± 115.6	a	604.3 ± 144.7	b	711.7 ± 167	a
Fe in liver [µg]	R	2194 ± 394	a	1421 ± 245	b	1642 ± 244	b
	B	2132 ± 58	a	1652 ± 247	b	1734 ± 297	b
Fe in blood [µg/dL]	R	126 ± 13.5	a	140.5 ± 24.1	b	115 ± 14.9	a
	B	123.8 ± 18.9	a	160 ± 27.4	b	100.3 ± 10.6	a

a, b indicate statistical differences between additives (Without, M, Om) *p* < 0.05. * indicate statistical differences between types of bread (R, B) *p* < 0.05.

**Table 4:** Zinc content in organs.

Ingredients	Type of Bread	Without		M		Om	
Zn in consumed diet [g]	R	17.58 ± 1.79	a *	21.55 ± 1.41	b *	20.89 ± 1.39	b *
	B	21.48 ± 2.48	a *	30.48 ± 2.77	b *	28.89 ± 2.67	b *
Zn in femur [µg/g d.m.]	R	711.7 ± 125.8	a *	446.3 ± 38.9	b *	454.3 ± 35.3	b
	B	520.4 ± 56.6	a *	510.7 ± 21.3	a *	487.3 ± 78.3	a
Zn in femur [µg]	R	301 ± 22.1	a	227.7 ± 15.6	b	225.4 ± 12.9	b
	B	245 ± 33.8	a	251.1 ± 26.1	a	239.6 ± 29.6	a
Zn in kidney [µg/g d.m.]	R	119.6 ± 10.2	a	110.3 ± 6.5	a	109.7 ± 9.3	a
	B	125.8 ± 14.8	a	115 ± 8	a	110.7 ± 4.8	a
Zn in kidney [µg]	R	54.58 ± 6.36	a	59.71 ± 5.23	a	56.82 ± 6.87	a
	B	62.39 ± 5.75	a	62.64 ± 3.4	a	60.45 ± 6.69	a
Zn in spleen [µg/g d.m.]	R	116 ± 11	a	122.2 ± 10	a	127 ± 7.8	a
	B	138.6 ± 17.5	a	114.1 ± 4.5	a	119.7 ± 17	a
Zn in spleen [µg]	R	13.75 ± 1.55	a	13.99 ± 0.84	a	13.82 ± 0.86	a
	B	13.99 ± 1.26	a	14.42 ± 0.93	a	13.16 ± 1.12	a
Zn in heart [µg/g d.m.]	R	93.4 ± 6.39	a	83.56 ± 13.32	a *	107.52 ± 32.67	a
	B	83.08 ± 12.19	a	165.59 ± 46.11	b *	84.21 ± 2.91	a
Zn in heart [µg]	R	20.49 ± 1.05	a	20.72 ± 1.18	a	20.19 ± 1.22	a
	B	20.03 ± 0.93	a	19.96 ± 0.88	a	19.84 ± 1.13	a
Zn in liver [µg/g d.m.]	R	124.7 ± 6.6	a *	69.5 ± 13.7	b *	95.6 ± 28.6	a
	B	98.6 ± 24.7	a *	93.6 ± 17	a *	107.2 ± 34.1	a
Zn in liver [µg]	R	284 ± 35.6	a	197.7 ± 17.5	b	250.6 ± 101.3	a
	B	268.5 ± 48.6	a	259.8 ± 50.7	a	250.5 ± 74.4	a
Zn in blood [µmol/L]	R	15.3 ± 0.46	a	17.62 ± 1.09	b	15.18 ± 1.37	a
	B	14.38 ± 0.99	a	16.98 ± 1.73	b	15.33 ± 2.56	a
Zn in blood [µg/L]	R	999.1 ± 29.9	a	1150.6 ± 71.4	b	991.3 ± 89.5	a
	B	938.7 ± 64.7	a	1108.8 ± 112.7	a	1000.7 ± 166.9	a

a, b indicate statistical differences between additives (Without, M, Om) *p* < 0.05. * indicate statistical differences between types of bread (R, B) *p* < 0.05.

**Table 5:** Copper content in organs.

Ingredients	Type of Bread	Without		M		Om	
Cu in consumed diet [g]	R	2.72 ± 0.28	a *	4.01 ± 0.26	b	3.87 ± 0.26	b
	B	3.39 ± 0.28	a *	4.51 ± 0.41	b	4.14 ± 0.38	b
Cu in femur µg/g d.m.]	R	6.86 ± 0.77	a *	5.01 ± 0.45	b	5.22 ± 0.62	b
	B	5.21 ± 0.41	a *	5.03 ± 0.62	a	5.29 ± 0.77	a
Cu in femur [µg]	R	2.92 ± 0.13	a *	2.51 ± 0.26	b	2.58 ± 0.43	b
	B	2.53 ± 0.14	a *	2.5 ± 0.32	a	2.61 ± 0.38	a
Cu in kidney [µg/g d.m.]	R	21.72 ± 1.52	a	26.5 ± 1.61	b	22.16 ± 1.94	a
	B	21.64 ± 2.45	a	28.5 ± 2.44	b	20.81 ± 1.63	a
Cu in kidney [µg]	R	9.93 ± 1.32	a	15.06 ± 1.07	b	11.26 ± 1.15	b
	B	10.79 ± 1.56	a	14.78 ± 1.81	a	11.32 ± 1.06	b
Cu in spleen [µg/g d.m.]	R	5.05 ± 0.6	a *	5.65 ± 1.91	a	10.04 ± 1.63	b
	B	9.04 ± 1.16	a *	6.09 ± 1.52	b	9.95 ± 3	a
Cu in spleen [µg]	R	0.594 ± 0.058	a *	0.652 ± 0.239	a	1.052 ± 0.193	b
	B	0.88 ± 0.104	a *	0.711 ± 0.213	a	1.098 ± 0.319	b
Cu in heart [µg/g d.m.]	R	24.95 ± 0.88	a *	22.43 ± 3.52	a *	30.8 ± 9.59	b
	B	23.4 ± 4.28	a *	48.68 ± 11.59	b *	25.3 ± 2.4	a
Cu in heart [µg]	R	5.02 ± 0.6	a	5.68 ± 0.71	a	5.52 ± 0.6	a
	B	5.45 ± 0.82	a	6.03 ± 0.31	a	5.57 ± 0.39	a
Cu in liver [µg/g d.m.]	R	28.95 ± 3.47	a *	18.5 ± 3.65	b	21.32 ± 5.12	b
	B	21.81 ± 3.84	a *	20.13 ± 3.18	a	17.89 ± 6.68	a
Cu in liver [µg]	R	65.61 ± 7.12	a	50.79 ± 9.06	a	51.76 ± 16.17	a
	B	59.46 ± 4.77	a	55.11 ± 10.43	a	48.51 ± 14.68	a
Cu in blood [µg/L]	R	1121 ± 18	a *	1171 ± 102	a	1217 ± 59	a
	B	1332 ± 183	a *	1246 ± 256	a	1248 ± 123	a

a, b indicate statistical differences between additives (Without, M, Om) *p* < 0.05. * indicate statistical differences between types of bread (R, B) *p* < 0.05.

**Table 6:** The content of minerals in organs in relation to its consumption.

Ingredients		Type of Bread	Without		M		Om	
Fe	in femur/intake [µg/g]	R	2.77 ± 0.6	a	2.45 ± 0.31	a *	1.86 ± 0.23	b
		B	2.41 ± 0.29	a	1.69 ± 0.24	b *	1.91 ± 0.27	ab
	in kidney/intake [µg/g]	R	6.76 ± 1.52	a	5.22 ± 0.41	a	3.98 ± 0.59	b
		B	4.78 ± 1.15	a	4.25 ± 0.76	a	3.73 ± 0.54	a
	in spleen/intake [µg/g]	R	12,49 ± 3.98	a *	6.3 ± 1.02	b	6.81 ± 1.18	b *
		B	7.07 ± 1.45	a *	7.07 ± 1.76	a	9.02 ± 0.99	b *
	in heart/intake [µg/g]	R	3.31 ± 0.35	a *	2.78 ± 0.24	ab *	2.67 ± 0.14	b *
		B	2.76 ± 0.16	a *	2.21 ± 0.23	b *	2.29 ± 0.25	b *
	in liver/intake [µg/g]	R	114.2 ± 20.7	a *	55.9 ± 8.8	b	63.7 ± 12.3	b
		B	86.7 ± 9.7	a *	55.5 ± 10.2	b	58.4 ± 13.2	b
	in blood/intake [µg/dl/g]	R	6.55 ± 0.62	a	5.57 ± 0.95	a	4.41 ± 0.72	b *
		B	5.01 ± 0.73	a	5.28 ± 0.75	a	3.26 ± 0.5	b *
Zn	in femur/intake [µg/g]	R	17.64 ± 1.93	a *	10.34 ± 0.44	b *	10.74 ± 0.42	b *
		B	11.49 ± 1.68	a *	8.26 ± 0.73	b *	8.33 ± 1.03	b *
	in kidney/intake [µg/g]	R	3.1 ± 0.1	a	2.77 ± 0.11	b *	2.71 ± 0.18	b *
		B	2.91 ± 0.13	a	2.02 ± 0.17	b *	2.09 ± 0.06	b *
	in spleen/intake [µg/g]	R	0.782 ± 0.044	a *	0.651 ± 0.052	b *	0.663 ± 0.05	b *
		B	0.654 ± 0.038	a *	0.476 ± 0.043	b *	0.456 ± 0.021	b *
	in heart/intake [µg/g]	R	1.18 ± 0.18	a	0.97 ± 0.13	b *	0.97 ± 0.11	b *
		B	0.95 ± 0.15	a	0.62 ± 0.02	b *	0.69 ± 0.09	b *
	in liver/intake [µg/g]	R	16.24 ± 2.23	a *	9.28 ± 0.73	b	12.45 ± 4.92	c
		B	0.654 ± 0.038	a *	0.476 ± 0.043	b *	0.456 ± 0.021	b *
	in blood/intake [µg/dl/g]	R	58.76 ± 7.8	a *	53.19 ± 6.63	a *	48.13 ± 6.11	a *
		B	43.53 ± 5.12	a *	36.06 ± 6.38	a *	34.42 ± 7.52	a *
Cu	in femur/intake [µg/g]	R	1.11 ± 0.16	a *	0.62 ± 0.05	b	0.66 ± 0.08	b
		B	0.75 ± 0.08	a *	0.54 ± 0.06	b	0.63 ± 0.1	b
	in kidney/intake [µg/g]	R	3.65 ± 0.12	a	3.68 ± 0.16	a	2.91 ± 0.24	ab
		B	3.29 ± 0.26	a	3.3 ± 0.47	a	2.74 ± 0.1	b
	in spleen/intake [µg/g]	R	0.226 ± 0.002	a	0.164 ± 0.073	b	0.264 ± 0.036	c
		B	0.271 ± 0.044	a	0.155 ± 0.048	b	0.265 ± 0.075	a
	in heart/intake [µg/g]	R	1.85 ± 0.08	a *	1.42 ± 0.13	b	1.42 ± 0.13	b
		B	1.68 ± 0.08	a *	1.27 ± 0.06	b	1.35 ± 0.09	b
	in liver/intake [µg/g]	R	24.48 ± 4.73	a *	12.79 ± 1.84	b	13.82 ± 4.87	b
		B	17.36 ± 1.21	a *	12.4 ± 2.22	b	11.85 ± 4.54	b
	in blood/intake [µg/dL/g]	R	425.4 ± 44.3	a *	292.8 ± 26.7	b	316 ± 28.5	c
		B	381.4 ± 42.6	a *	281.5 ± 79.4	b	297.1 ± 33.4	b

a, b, c indicate statistical differences between additives (Without, M, Om) *p* < 0.05. * indicate statistical differences between type of bread (R, B) *p* < 0.05.

## References

[1] Aguilar N., Albanell E., Miñarro B., Capellas M. (2016). Chestnut Flour Sourdough for Gluten-Free Bread Making. Eur. Food Res. Technol..

[2] Blanco C.A., Ronda F., Pérez B., Pando V. (2011). Improving Gluten-Free Bread Quality by Enrichment with Acidic Food Additives. Food Chem..

[3] Allen B., Orfila C. (2018). The Availability and Nutritional Adequacy of Gluten-Free Bread and Pasta. Nutrients.

[4] Barera G., Beccio S., Proverbio M.C., Mora S. (2004). Longitudinal Changes in Bone Metabolism and Bone Mineral Content in Children with Celiac Disease During Consumption of a Gluten-Free Diet. Am. J. Clin. Nutr..

[5] Bascunan K.A., Vespa M., Araya M. (2017). Celiac Disease: Understanding the Gluten-Free Diet. Eur. J. Nutr..

[6] Bourekoua H., Rozylo R., Gawlik-Dziki U., Benatallah L., Zidoune M.N., Dziki D. (2018). Pomegranate Seed Powder as a Functional Component of Gluten-Free Bread (Physical, Sensorial and Antioxidant Evaluation). Int. J. Food Sci. Technol..

[7] Gujral H.S., Guardiola I., Carbonell J.V., Rosell C.A. (2003). Effect of Cyclodextrinase on Dough Rheology and Bread Quality from Rice Flour. J. Agric. Food Chem..

[8] Elli L., Pigatto P.D., Guzzi G. (2018). Evaluation of Metals Exposure in Adults Ona Gluten-Free Diet. Clinical gastroenterology and hepatology. Off. Clin. Pract. J. Am. Gastroenterol. Assoc..

[9] Raehsler S.L., Choung R.S., Marietta E.V., Murray J.A. (2018). Accumulation of Heavy Metals in People on a Gluten-Free Diet. Clin. Gastroenterol. Hepatol..

[10] Suliburska J., Krejpcio Z. (2014). Evaluation of the Content and Bioaccessibility of Iron, Zinc, Calcium and Magnesium from Groats, Rice, Leguminous Grains and Nuts. J. Food Sci. Technol. Mysore.

[11] Bjorklund G., Aaseth J., Skalny A.V., Suliburska J., Skalnaya M.G., Nikonorov A.A., Tinkov A.A. (2017). Interactions of Iron with Manganese, Zinc, Chromium, and Selenium as Related to Prophylaxis and Treatment of Iron Deficiency. J. Trace Elem. Med. Biol..

[12] Regula J., Cerba A., Suliburska J., Tinkov A.A. (2018). In Vitro Bioavailability of Calcium, Magnesium, Iron, Zinc, and Copper from Gluten-Free Breads Supplemented with Natural Additives. Biol. Trace Elem. Res..

[13] Brown K.H., Peerson J.M., Rivera J., Allen L.H. (2002). Effect of Supplemental Zinc on the Growth and Serum Zinc Concentrations of Prepubertal Children: A Meta-Analysis of Randomized Controlled Trials. Am. J. Clin. Nutr..

[14] Della Lucia C.M., Santos L.L.M., Rodrigues K.C.D., Rodrigues V.C.D., Martino H.S.D., Sant’Ana H.M.P. (2014). Bioavailability of Zinc in Wistar Rats Fed with Rice Fortified with Zinc Oxide. Nutrients.

[15] Rogaska A., Regula J., Krol E. (2020). The Effect of Diets Including Gluten-Free Bread Enriched with Natural Additives on the Content of Calcium and Magnesium in Rats with Deficiencies of These Elements. J. Elem..

[16] Rahaie S., Gharibzahedi S.M.T., Razavi S.H., Jafari S.M. (2014). Recent Developments on New Formulations Based on Nutrient-Dense Ingredients for the Production of Healthy-Functional Bread: A Review. J. Food Sci. Technol. Mysore.

[17] Campo E., del Arco L., Urtasun L., Oria R., Ferrer-Mairar A. (2016). Impact of Sourdough on Sensory Properties and Consumers’ Preference of Gluten-Free Breads Enriched with Teff Flour. J. Cereal Sci..

[18] Reeves P.G. (1997). Components of the Ain-93 Diets as Improvements in the Ain-76a Diet. J. Nutr..

[19] Suliburska J., Bogdanski P., Jakubowski H. (2014). The Influence of Selected Antihypertensive Drugs on Zinc, Copper, and Iron Status in Spontaneously Hypertensive Rats. Eur. J. Pharmacol..

[20] Suliburska J., Krejpcio Z., Reguła J., Grochowicz A. (2013). Evaluation of the Content and the Potential Bioavailability of Minerals from Gluten-Free Products. Acta Sci. Pol. Technol. Aliment..

[21] Ghafoor K., Ozcan M.M., Al-Juhaimi F., Babiker E.E., Fadimu G.J. (2019). Changes in Quality, Bioactive Compounds, Fatty Acids, Tocopherols, and Phenolic Composition in Oven- and Microwave-Roasted Poppy Seeds and Oil. Lwt Food Sci. Technol..

[22] Horstmann S.W., Atzler J.J., Heitmann M., Zannini E., Lynch K.M., Arendt E.K. (2019). A Comparative Study of Gluten-Free Sprouts in the Gluten-Free Bread-Making Process. Eur. Food Res. Technol..

[23] Hosseini S.M., Soltanizadeh N., Mirmoghtadaee P., Banavand P., Mirmoghtadaie L., Shojaee-Aliabadi S. (2018). Gluten-Free Products in Celiac Disease: Nutritional and Technological Challenges and Solutions. J. Res. Med. Sci..

[24] Swieca M., Regula J., Suliburska J., Zlotek U., Gawlik-Dziki U., Ferreira I. (2020). Safeness of Diets Based on Gluten-Free Buckwheat Bread Enriched with Seeds and Nuts-Effect on Oxidative and Biochemical Parameters in Rat Serum. Nutrients.

[25] Rosado J.L. (2003). Zinc and Copper: Proposed Fortification Levels and Recommended Zinc Compounds. J. Nutr..

